# Serum thyroglobulin reference intervals in regions with adequate and more than adequate iodine intake

**DOI:** 10.1097/MD.0000000000005273

**Published:** 2016-12-02

**Authors:** Zhaojun Wang, Hanyi Zhang, Xiaowen Zhang, Jie Sun, Cheng Han, Chenyan Li, Yongze Li, Xiaochun Teng, Chenling Fan, Aihua Liu, Zhongyan Shan, Chao Liu, Jianping Weng, Weiping Teng

**Affiliations:** a The Endocrine Institute and The Liaoning Provincial Key Laboratory of Endocrine Diseases, Department of Endocrinology and Metabolism, The First Hospital of China Medical University, Liaoning; b Department of Endocrinology and Metabolism, Jiangsu Province Hospital on Integration of Chinese and Western Medicine, Nanjing University of Traditional Chinese Medicine, Jiangsu; c Guangdong Provincial Key Laboratory of Diabetology, Department of Endocrinology and Metabolic Disease, The Third Affiliated Hospital of Sun Yat-Sen University, Guangdong, China.

**Keywords:** iodine intake, normal range, reference interval, thyroglobulin, TSH, UIC

## Abstract

The purpose of this study was to establish normal thyroglobulin (Tg) reference intervals (RIs) in regions with adequate and more than adequate iodine intake according to the National Academy of Clinical Biochemistry (NACB) guidelines and to investigate the relationships between Tg and other factors.

A total of 1317 thyroid disease-free adult subjects (578 men, 739 nonpregnant women) from 2 cities (Guangzhou and Nanjing) were enrolled in this retrospective, observational study. Each subject completed a questionnaire and underwent physical and ultrasonic examination. Serum Tg, thyroid-stimulating hormone (TSH), thyroid peroxidase antibody (TPOAb), Tg antibody (TgAb), and urinary iodine concentration (UIC) were measured. Reference groups were established on the basis of TSH levels: 0.5 to 2.0 and 0.27 to 4.2 mIU/L.

The Tg RIs for Guangzhou and Nanjing were 1.6 to 30.0 and 1.9 to 25.8 ng/mL, respectively. No significant differences in Tg were found between genders or among different reference groups. Stepwise linear regression analyses showed that TgAb, thyroid volume, goiter, gender, age, and TSH levels were correlated with Tg.

In adults from regions with adequate and more than adequate iodine intake, we found that Tg may be a suitable marker of iodine status; gender-specific Tg RI was unnecessary; there was no difference between Tg RIs in regions with adequate and more than adequate iodine intake; and the TSH criterion for selecting the Tg reference population could follow the local TSH reference rather than 0.5 to 2.0 mIU/L.

## 1 Introduction

Thyroglobulin (Tg) is the most abundant protein in the thyroid gland. It is produced by thyroid follicular cells, iodized as active thyroid hormone, and released into circulation through exocytosis.^[1]^ Clinicians use the serum concentration of Tg to monitor thyroid cancer^[2–4]^; several studies have suggested using it as a biomarker of iodine status at both the individual and population level,^[5–7]^ which requires a region-specific Tg reference. The National Academy of Clinical Biochemistry (NACB) has proposed guidelines for establishing such a reference^[8]^; however, very few studies refer to this guideline. For this reason, the present study established proper Tg reference intervals (RIs) for 2 regions within China that have different iodine statuses.

## 2 Methods

We performed a retrospective, observational study from 2010 to 2011; 1343 thyroid disease free adult residents of 2 cities, Guangzhou and Nanjing, were enrolled in the current study. Guangzhou represented adequate iodine intake and Nanjing represented more than adequate iodine intake.

The Guangzhou participants were selected to be the reference group using the following criteria according to the NACB guidelines^[8]^:

(1) Age ≥18 but ≤40 years.
(2) Thyroid-stimulating hormone (TSH) >0.5 mIU/L but <2.0 mIU/L.
(3) Nonsmoker (including passive smoker).
(4) No personal or family history of thyroid diseases.
(5) No use of drugs that might influence iodine metabolism.
(6) No goiter detection upon physical examination or ultrasonography.
(7) Testing negative for thyroid autoantibodies [Tg antibody (TgAb) <115 IU/mL, thyroid peroxidase antibody (TPOAb) <34 IU/mL].

This group was defined as Guangzhou reference group A (TSH 0.5–2.0 mIU/L, n = 160). Another reference group was designed in accordance with these criteria, except that TSH levels differed (0.27–4.2 mIU/L, RI provided by Cobas Eless 601, Roche Diagnostics Ltd, Switzerland), and was defined as Guangzhou reference group B (TSH 0.27–4.2 mIU/L, n = 314). Nanjing reference group A (TSH 0.5–2.0 mIU/L, n = 54) and Nanjing reference group B (TSH 0.27–4.2 mIU/L, n = 160) were similarly defined.

All of the subjects completed questionnaires covering pertinent information such as age, gender, smoking, drug intake, and any personal or family history of thyroid conditions.

Clinical diagnosis of goiter was performed by physical examination and ultrasonography (model SA600 with 7.5MHz linear transducer, Medison Co. Ltd., South Korea). The goiter was defined as a visible or palpable thyroid nodule as well as a nodule appearing on the thyroid ultrasound, or a thyroid volume (TV) >25.6 mL for men and >19.4 mL for women.^[9]^

Blood and spot urine samples were obtained from each participant at 8.00 to 10.00 am after an overnight fast. All of the specimens were stored at -80°C until analysis. UICs were measured using an ammonium persulfate method based on the Sandell–Kolthoff reaction. The intra- and interassay coefficients of variation (CV) were 3% to 4% and 4% to 6% at 66 μg/L, respectively, and 2% to 5% and 3% to 6% at 230 μg/L, respectively. Serum Tg was measured on a Roche Cobas Eless 601 module immunology analyzer (Switzerland) using the electrochemiluminescence immunoassay (ECLIA) method, which was standardized against the human Tg reference material (CRM 457). This method had a LoB (limit of blank), LoD (limit of detection), and LoQ (limit of quantification) of 0.02, 0.04, and 0.1 ng/mL, respectively; the intra- and interassay CV values were 2.0% to 4.8% and 4.0% to 5.9%, respectively. Serum TSH, TPOAb, and TgAb were also measured using the ECLIA method. The intra-assay CV values were 1.57% to 4.12%, 2.42% to 5.63%, and 1.3% to 4.9%, respectively, whereas the interassay CV values were 1.26% to 5.76%, 5.23% to 8.16%, and 2.1% to 6.9%, respectively.

### 2.1 Statistical analysis

Data were recorded using Office Excel 2007, Microsoft Co. Ltd., and analyzed using SPSS 21.0, IBM Co. Ltd.; R software 3.3.1 was used for assessing the normality of the data distribution and false discovery rate (FDR) control. Data are expressed as mean ± standard deviation (SD). The Tg RI was calculated using the logarithmic transformed Tg value as the mean ± 1.96 SD and then transformed back. Tg outliers were checked using Chauvenet criterion. A bootstrap principle-based robust method was used for reference groups consisting of fewer than 120 subjects.^[10,11]^ A standard normal deviate test was used to determine whether a gender-specific RI should be calculated.^[12,13]^ The Anderson–Darling method was used to assess the normality of the data distribution. Spearman rho and linear regression were calculated to evaluate correlations between Tg and other factors. To test for differences in parameters among groups, Chi-square, Mann–Whitney *U* test, and 1-way analysis of variance (ANOVA; with Welch correction if unequal variances occurred) were used. Significance was set at *P* (or a FDR q value) <0.05.

### 2.2 Ethical aspects

All of the procedures involving human participants were performed in accordance with the ethical standards of the China Medical University and the 1964 Helsinki declaration, as well as its later amendments and comparable ethical standards. The research protocols were carefully explained. Informed consent was obtained from all individual participants included in the study.

## 3 Results

### 3.1 Subject characteristics and Tg distribution

A total of 1343 subjects participated in this study, of whom 26 individuals were excluded: 14 had Tg <0.1 ng/mL (LoQ), 8 had Tg >500 ng/mL (upper LoD), and 4 with missing data. A total of 1157 subjects (515 men, 642 women, aged 44.9 ± 15.2 years) from Guangzhou and 160 subjects (63 men, 97 women, aged 29.1 ± 6.4 years) from Nanjing were enrolled. The clinical characteristics of different groups are summarized in Table 1.

**Table 1:**
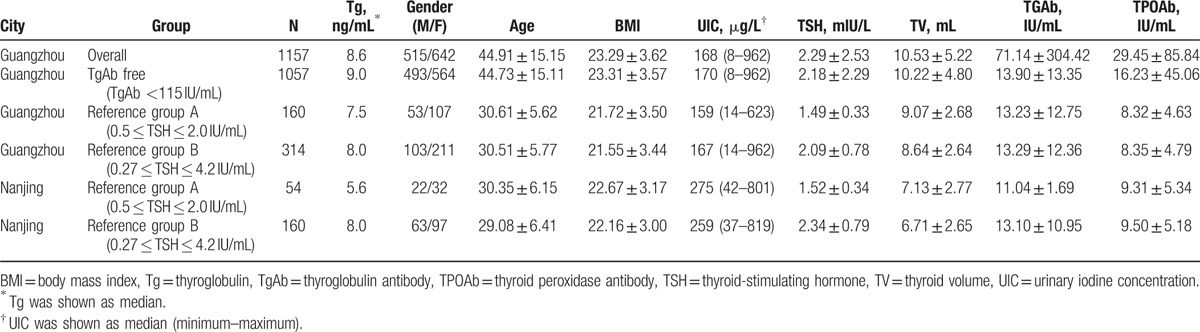
Clinical characteristics of different groups.

The distribution of Tg in each group exhibited a long-tailed positive skewness and kurtosis. The original Tg value was transformed as lg(Tg+1), which then became normally distributed. The Tg median of Guangzhou reference groups A and B was 7.5 and 8.0 ng/mL, respectively, and that of Nanjing reference groups A and B was 5.6 and 8.0 ng/mL, respectively. There were no significant differences in age (*P* *=* 0.091) and gender (*P* *=* 0.385) among these 4 reference groups.

In the Guangzhou population, 78 of 515 men and 137 of 642 women were classified as iodine deficient according to World Health Organization (WHO) standards (i.e., having an UIC below 100 μg/L). Women had a higher proportion of iodine deficiency than men (*P* *=* 0.03).

### 3.2 Tg RIs in different subgroups

**Figure 1:**
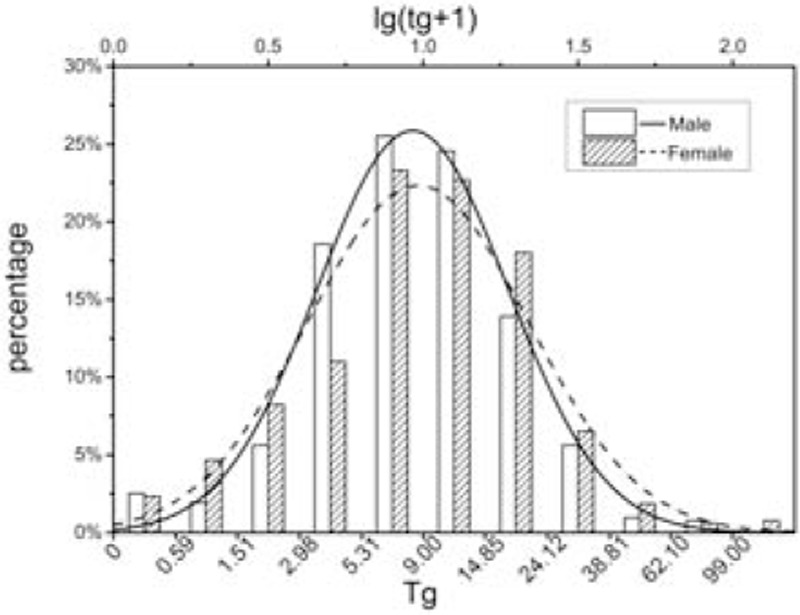
Tg distribution of men (n = 517) and women (n = 644) from Guangzhou. No difference was found between genders.

Before calculating the Tg RI, we tested for gender discrepancy in Tg distribution. In all 5 levels of grouping, that is, Guangzhou overall, Guangzhou reference group A and B and Nanjing reference group A and B, no significant differences in Tg were observed between genders (Mann–Whitney *U* test, *P* *=* 0.373, 0.418, 0.167, 0.793, 0.68, respectively) (Fig. 1). The ratio of SDs of the Guangzhou reference group A was <1.5 (1.03), and in the standard normal deviate test, *z* (1.40) was <*z*∗ (3.43). The other 3 reference groups also showed this characteristic; thus, determining gender-specific Tg RI was found to be unnecessary. Tg RI was calculated using the transformed Tg value as the mean ± 1.96 SD and then transformed back (Table 2).

**Table 2:**
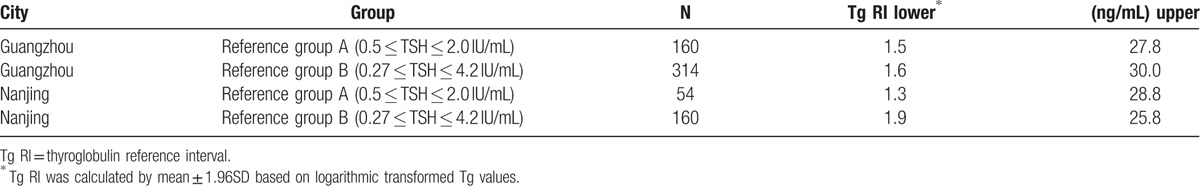
Thyroglobulin reference intervals calculated by different groups.

For Guangzhou, the RIs calculated by reference groups A (n = 160) and B (n = 314) were 1.5 to 27.8 and 1.6 to 30.0 ng/mL, respectively. There was no difference between these 2 RIs, as determined by either the Mann–Whitney *U* test using the original Tg value (*P* *=* 0.304), or by the *t* test using the transformed Tg value (*P* *=* 0.336). For this reason, the latter RI (i.e., 1.6–30.0 ng/mL) calculated by reference group B was used, because it contained nearly 2-fold more subjects than reference group A, making it more reliable.

For Nanjing, the Tg RIs calculated using reference groups A (n = 54) and B (n = 160) were 1.3 to 28.8 and 1.9 to 25.8 ng/mL, respectively, with no differences between them. The Tg RI calculated using reference group B was also used (i.e., 1.9–25.8 ng/mL).

### 3.3 Relationship between Tg and other factors

Subjects from Guangzhou were used for testing correlations between Tg and other factors. Because serum TgAb interferes with Tg immunoassays,^[14]^ subjects who tested positive for TgAb (>115 IU/mL) were excluded (final N = 1057). Correlations between Tg and other parameters were tested using the Spearman rank correlation test with FDR q-value correction. A multivariate linear regression model was also used to evaluate the relative impact of other coefficients on Tg.

In the Spearman correlation test, goiter (*P* *<* 0.001), age (*P* *<* 0.001), TV (*P* *<* 0.01), gender (*P* *=* 0.014), body mass index (BMI) (*P* *=* 0.019), and UIC (*P* *=* 0.029) were found to be associated with Tg. Moreover, no relationship was observed between Tg and smoking, iodide-containing drug intake, TSH, TPOAb, or TgAb. In stepwise linear regression, the model had an overall adjusted *R*-squared value of 0.096. TgAb (β = -0.157, *P* *<* 0.001), TV (β = 0.158, *P* *<* 0.001), goiter (β = 0.124, *P* *<* 0.001), gender (β = 0.111, *P* *<* 0.001), age (β = 0.095, *P* *=* 0.003), and TSH (β = 0.068, *P* *=* 0.02) were found to be associated with Tg. BMI, smoking, iodide-containing drug intake, UIC, and TPOAb were excluded from the model.

## 4 Discussion

This study presents the Tg RIs for 2 parts of China with different iodine statuses. The methods for Tg measurement have changed over the past several decades, and currently include radioimmunoassay (RIA),^[15–19]^ immunoradiometric assay (IRMA),^[19–23]^ immunoluminometric assay (ILMA), immunochemiluminescence assay (ICMA),^[24–28]^ enzyme-linked immunosorbent assay (ELISA),^[29,30]^ and mass spectrometry assay.^[31–33]^ Among these methods, interassay variation remains the principal challenge, as results can vary from 43% to 65% in healthy subjects.^[24,34]^ Using the BCR 457 Reference Material (formerly named CRM 457) only reduces interassay variation by about 14% to 27%^[6,35,36]^; therefore, it is important to develop local Tg RI.

Many studies have measured Tg in adults^[9,20,37,38–51]^; however, very few of them have provided information on Tg RI (Table 3). Iervasi et al^[47]^ reported a Tg upper reference limit (95th percentile) of 46.9 ng/mL (Tg median: 11.2 ng/mL) in 120 healthy TgAb-negative euthyroid subjects with no histories of thyroid diseases or abnormalities on thyroid ultrasonography. Bílek et al^[51]^ recruited a large population (aged 6–98 years, 1696 men, 2420 women) with a median Tg of 17.5 ng/mL, and suggested 44.2 ng/mL as the Tg upper limits. Feldt et al^[38]^ established correlated reference values for age and gender (men ≤40 years: 36 ng/mL, >40 years: 44 ng/mL; women ≤40 years: 30 ng/mL, >40 years: 60 ng/mL) based on 143 normal subjects. Giovanella et al^[37]^ found higher serum Tg levels in women than in men (*P* *=* 0.0022), and provided gender-specific reference values (men: 1.40–29.2 ng/mL; women: 1.50–38.5 ng/mL). Among these 4 studies, 3 described participants with higher Tg levels, and only 1 study^[37]^ reported values similar to those reported here. These differences may actually be attributable to the methods. Luca et al^[37]^ and the present work both used the ECLIA method to measure Tg, which was different from the methods used by other studies (e.g., RIA in Feldt et al,^[38]^ ICMA in Iervasi et al,^[47]^ and ELISA in Bílek et al).^[51]^ This confirms the importance of locality in developing Tg RI.

**Table 3:**
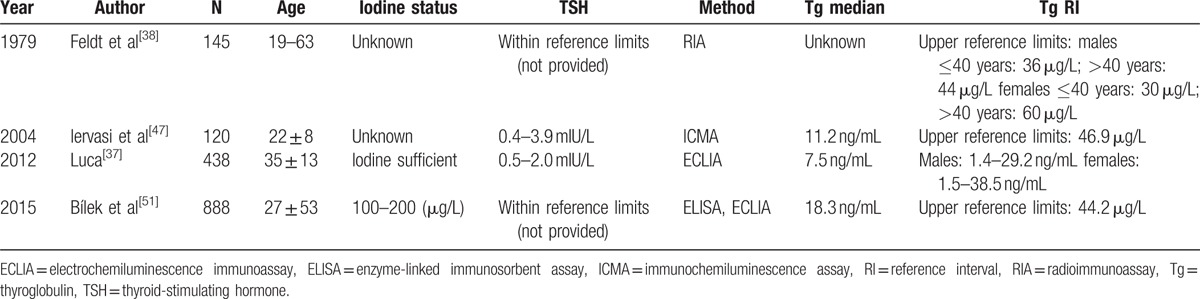
Previous studies calculating thyroglobulin reference intervals for adults.

Studies differ on whether gender-specific RI is required. Here, we hypothesized that gender-specific Tg RI is unnecessary, primarily for the following 3 reasons. First, in this study, although gender was significantly correlated with Tg (women > men, β = 0.111, *P* *=* 0.003), the correlation was too weak to show a significant difference between male and female subjects at all grouping levels. In addition, standard normal deviate testing did not justify establishing gender-specific Tg RI. Second, some studies that have reported gender differences in Tg failed to mention correction for other factors, which might have influenced Tg levels. Thus, the differences detected might actually be due to sampling strategies. Finally, as women were found to be more likely to have inadequate iodine intake, it is not inexplicable that women had higher Tg levels than men, resulting in a higher proportion of women exceeding the upper reference limit. Considering these facts, no gender-specific RI was developed for Tg.

In this study, Tg was correlated with TV, UIC, and TSH. Thus, Tg may serve as a good marker of iodine status. The median UIC of the 2 cities was 174 and 282 μg/L, respectively; however, there were no differences in Tg levels between the reference groups from the 2 regions with different iodine statuses. It was previously demonstrated that plotting Tg against UIC resulted in a “U”-shaped curve.^[5,24]^ The fact that these 2 regions have nearly the same Tg RI may actually reflect the well-documented “U”-shaped curve, and both median UICs were located at the bottom of the curve.

Another issue is the selection criterion of TSH for the Tg reference population. In the aforementioned studies that established Tg RI, only 1 study used a TSH level of 0.5 to 2.0 mUI/L, whereas the other studies used their own TSH reference levels. In this study, there was no difference between the Tg RIs calculated using the 2 reference groups (i.e., A and B) with different TSH values. For this reason, we suggest that using a reference group with a TSH value within a reasonable reference is acceptable. This greatly reduces the difficulty in subject selection, making it more feasible and commercial for Tg reference determination.

### 4.1 Limitations of the study

The current study had several limitations. First, the thyroid indexes did not include serum triiodothyronine (T_3_) or thyroxine (T_4_). Although the NACB guideline for Tg RI determination does not require these parameters, there may be certain individuals who have TSH levels within the reference values but abnormal T_3_ or T_4_ levels. Second, there were more women than men in this study (male:female ≈ 1:2), because in China, women are usually more concerned about health, and as such, are willing to participate in these type of studies. However, because no significant differences in Tg levels were found between genders, this gender-specific imbalance of Tg seemed to have minor effects on our conclusions. Finally, this is a cross-sectional study; thus, the causal relationship between Tg and iodine intake could not be well established.

## 5 Conclusion

Tg may be a useful marker of iodine status. Tg values are dependent on gender, but gender-specific Tg RI may be unnecessary. UIC correlates with Tg levels, but no differences exist between Tg RI in regions of adequate and more than adequate iodine intake. Finally, for the sake of simplifying subject selection for Tg RI, the TSH criterion can follow local TSH RIs other than values of 0.5 to 2.0 mIU/L.

## Acknowledgment

We gratefully acknowledge the contribution of physicians from Guangzhou and Nanjing. We would also like to thank the residents who participated in this study.
